# Comparative analysis identifies micro‐RNA associated with nutrient homeostasis, development and stress response in *Arabidopsis thaliana* upon high Zn and metal hyperaccumulator *Arabidopsis halleri*

**DOI:** 10.1111/ppl.13488

**Published:** 2021-07-05

**Authors:** Elisa Fasani, Giovanni DalCorso, Gianluca Zorzi, Nicola Vitulo, Antonella Furini

**Affiliations:** ^1^ Department of Biotechnology University of Verona Verona Italy

**Keywords:** *Arabidopsis halleri*, *Arabidopsis thaliana*, development, metal hyperaccumulation, miRNA, nutrition, stress, Zn homeostasis

## Abstract

miRNAs have been found to be key players in mineral homeostasis, both in the control of nutrient balance and in the response to toxic trace elements. However, the effect of Zn excess on miRNAs has not been elucidated; moreover, no data are present regarding miRNAs in hyperaccumulator species, where metal homeostasis is tightly regulated. Therefore, expression levels of mature miRNAs were measured by RNA‐Seq in Zn‐sensitive *Arabidopsis thaliana* grown in control conditions and upon high Zn, in soil and in Zn‐hyperaccumulator *Arabidopsis halleri* grown in control conditions. Differential expression of notable miRNAs and their targets was confirmed by real‐time RT‐PCR. The comparison in *A. thaliana* revealed a small subset modulated upon Zn treatment that is associated with stress response and nutrient homeostasis. On the other hand, a more consistent group of miRNAs was differentially expressed in *A. halleri* compared with *A. thaliana*, reflecting inherent differences in nutritional requirements and response to stresses and plant growth and development. Overall, these results confirm the involvement of miRNAs in Zn homeostasis and support the hypothesis of distinct regulatory pathways in hyperaccumulator species.

## 1 INTRODUCTION

Micro‐RNAs (miRNAs) are small single‐stranded noncoding RNAs involved in regulating gene expression by repression of specific targets. Their biogenesis in plants proceeds by a complex pathway that ensures flexibility and adaptability to endogenous and environmental stimuli; such processes have been reviewed extensively (Achkar et al. 2016; Song et al. 2019). miRNA activity can regulate gene expression in different ways. These include (1) direct cleavage and subsequent degradation of target mRNA, (2) translational repression by blocking ribosome recruitment and/or progression, and (3) direction of DNA methylation (Song et al. 2019). To add to the complexity of the system, both miRNA transcription and processing and their activity are tightly regulated (Song et al. 2019; Stepien et al. 2017).

miRNAs are involved in all plant processes, including metabolism and development, interaction with the environment and stress responses. In particular, they are key players in determining phenotypic plasticity in response to environmental stimuli, such as light, temperature and nutrient availability. Regarding stresses, they are involved in plant immunity toward a variety of pathogens, as well as in response to different abiotic stresses (Song et al. 2019). Indeed, modulation of large sets of miRNAs during abiotic stress response has been demonstrated for extreme temperatures (Chen et al. 2012; Yu et al. 2012; Zeng et al. 2018; Zhang et al. 2014a, 2014b), drought (Bertolini et al. 2013; Zhang et al. 2014a, 2014b), salinity (Carnavale Bottino et al. 2013; Sun et al. 2015), and wounding (Tang et al. 2012; Wang et al. 2014). In addition to miRNA transcriptional control, the whole biogenetic machinery responds to stress conditions through transcriptional, posttranslational and proteolytic regulation on several elements of the biogenetic complexes (Manavella et al. 2019).

Among the environmental conditions triggering miRNA modulation, metal content in soil is a significant one. Indeed, plant homeostasis of trace elements is tightly regulated, as plants must both ensure adequate uptake and metabolism of essential elements and avoid toxicity due to excess of micronutrients as well as the presence of nonessential elements. In this context, miRNAs are key players in the fine‐tuning of metal homeostasis. For example, analyses in Cu‐deficient conditions revealed the up‐regulation of a highly conserved set of miRNAs including miR397, miR398, miR408, and miR857, leading to repression of dispensable Cu‐containing proteins and redistribution of Cu reserves toward essential processes such as photosynthesis (Abdel‐Ghany & Pilon 2008; Lu et al. 2011). On the other hand, Cu excess, an increasingly common situation due to agricultural practices, promotes down‐regulation of previously cited Cu‐responsive miRNAs, as well as modulation of others involved in stress response and metal transport (Fu et al. 2019; Jiu et al. 2019). Several toxic metals and metalloids have also been found to induce significant modulation of miRNAs. The involvement of the latter in stress due to Cd, Hg, As, Al, Pb, Cr, and Mn has been extensively reviewed (Ding et al. 2020; Noman et al. 2019; Noman & Aqeel 2017). In these cases, miRNA activity generally leads to reorganization of plant development and modulation of antioxidants and stress responses, although the modulated sets of miRNAs are element‐specific (Ding et al. 2020; Noman & Aqeel 2017).

Zn is an extremely interesting element in this context: indeed, as a micronutrient, Zn is essential for all living organisms. This metal is a fundamental co‐factor, with both structural and catalytic functions, in a large variety of proteins. It has been estimated that, on average, about 9% of the whole eukaryotic proteome is composed of Zn‐binding proteins, of the latter, the majority is predicted to be either enzymes (47%) or transcription factors (44%; Andreini et al. 2009). In *Arabidopsis thaliana*, more than 2000 proteins have been proposed by bioinformatic analysis to bind or transport Zn (Andreini et al. 2009), involved in a variety of extremely important processes, including DNA synthesis, transcription and translation, photosynthesis and proteolytic control of protein activity (Hänsch & Mendel 2009). On the other hand, Zn excess can prove detrimental for plants: toxicity can derive from competition with other metallic co‐factors and indirect formation of reactive oxygen species (ROS), resulting in impairment of photosynthesis, cell death and generally stunted growth (DalCorso 2012). In light of this, Zn homeostasis in plants needs to be under tight control. Although precise determination of Zn requirements is difficult to achieve, it has been estimated that the internal level of free Zn is below the nanomolar range in eukaryotic cells. In plants, optimal Zn concentrations, including both free and chelated or compartmentalized metal, are generally between 15 and 50 mg kg^−1^ dry biomass (Hänsch & Mendel 2009; Sinclair & Krämer 2012). However, this range is extremely variable, in line with the vast natural diversity of plants adapted to different environments and edaphic conditions.

Interestingly, a class of plants has been identified, called hyperaccumulators, able to accumulate extremely high metal concentrations in their above‐ground tissues and to tolerate them without showing toxicity symptoms (Baker & Whiting 2002; Krämer 2010). Among them, the facultative metallophyte *Arabidopsis halleri* is particularly interesting due to its constitutive ability to hyper accumulate Zn and its close phylogenetic proximity with model species *A. thaliana* (Krämer 2010). Transcriptomic analyses comparing nonaccumulator *A. thaliana* with hyperaccumulator *A. halleri* highlighted differential modulation of several genes involved in nutrient homeostasis and stress responses, many of which are constitutively expressed at high levels in *A. halleri* (Becher et al. 2004; Talke et al. 2006; Weber et al. 2004).

Despite the amount of data available for protein‐coding transcriptome, very little work has been produced regarding the Zn effect on small regulatory RNAs. Zn‐deficient conditions were analyzed in *Brassica juncea* and *Sorghum bicolor*, revealing a comparatively small set of modulated miRNAs, mostly involved in plant development and stress response (Li et al. 2013; Shi et al. 2013). However, no evidence is present concerning their modulation under Zn excess. Therefore, this study aims to investigate miRNA modulation in *A. thaliana* when treated with high Zn supplementation in soil. A transcriptomic analysis by miRNA‐Seq was performed comparing untreated and treated *A. thaliana* with untreated *A. halleri* plants to estimate both the Zn effect on the nonaccumulator species and naturally active strategies in the hyperaccumulator species. The results indicated a major variation in expression between the two species. Only a small miRNA subset was found to be differentially expressed in *A. thaliana* upon Zn treatment. Overall, modulated miRNAs participate mainly in the control of plant development, nutrient uptake and distribution, and stress response, confirming the involvement of small regulatory RNAs in controlling global plant processes associated with Zn homeostasis.

## 2 MATERIALS AND METHODS

### 2.1 Plant material and growth conditions

*Arabidopsis thaliana* (L.) Heynh. accession Columbia and *Arabidopsis halleri* (L.) O'Kane & Al‐Shehbaz population I16 (Val del Riso, northern Italy, 45°51034.40 N 9°52034.94 E; Meyer et al. 2015) were used for this study. Seeds were stratified for 3 days at 4°C to break seed dormancy, then sown in garden soil and grown in a growth chamber on a short‐day regime (8 hr light/16 hr dark, illumination 100–120 μmol m^−2^ s^−1^, day/night temperature 22/18°C). Four‐week‐old plants were watered with either water (*A. thaliana* control and *A. halleri*) or with 500 μM ZnSO_4_ (*A. thaliana* + Zn) for 1 week. Total Zn content in treated and untreated soil was measured at the end of the experiment by inductively coupled plasma atomic emission spectrometry (ICP‐AES), as described by Fasani et al. (2019). Total Zn in soil was 52.6 ± 1.1 mg kg^−1^ DW in control conditions and 165.0 ± 5.9 mg kg^−1^ DW in Zn‐treated conditions; these results are comparable with the mean values observed worldwide for Zn content in soils and with a moderately Zn‐rich soil, respectively (Alloway 2008; Kabata‐Pendias 1995). CaCl_2_‐extractable Zn, corresponding to soluble and exchangeable metal, was obtained by incubating air‐dried soil in 10 mM CaCl_2_ in a 1:2.5 proportion for 16 hr; the resulting solution was filtered and analyzed by ICP‐AES. CaCl_2_‐extractable Zn was 0.23 ± 0.01 mg kg^−1^ DW in control conditions and 0.75 ± 0.02 mg kg^−1^ DW in Zn‐treated conditions; these values fall in the range of CaCl_2_‐extractable Zn obtained in previous studies (Esnaola et al. 2000; Pueyo et al. 2004).

Rosettes of untreated and treated plants were collected and frozen in liquid nitrogen for further analyses. Three pools of five plants each were collected for each condition and genotype and considered as three different biological replicates.

### 2.2 Physiological analysis of plants

Chlorophylls were extracted in 80% aqueous acetone buffered with NaCO_3_; total chlorophyll content was measured as described by Porra et al. (1989).

In situ O_2_^−^ accumulation in above‐ground tissues was detected by nitroblue tetrazolium (NBT) staining, as reported in Rossetti and Bonatti (2001). Superoxide dismutase (SOD) activity was evaluated by native polyacrylamide gel electrophoresis (PAGE) and in‐gel NBT staining, as in Chu et al. (2005); equal protein loading (30 μg) was demonstrated by Coomassie staining of a replica gel in SDS‐PAGE. Quantification of global SOD activity was achieved by scanning the gels and determining band intensity with Quantity OneR software v4.4.1 (Bio‐Rad).

Zn concentration in leaves was determined by ICP‐AES as previously indicated (Fasani et al. 2019). Each of the analyses here reported was performed in triplicate.

### 2.3 miRNA‐Seq analysis

Sampled material was ground in liquid nitrogen, and small RNAs were extracted using the mirPremier microRNA Isolation Kit (Sigma‐Aldrich), according to the manufacturer's instructions. Small RNA concentration and purity were measured using a NanoDrop OneC Microvolume UV–Vis Spectrophotometer (Thermo Fisher Scientific), and integrity was assessed by the Bioanalyzer Small RNA Analysis Kit (Agilent Technologies) and the 2100 Bioanalyzer (Agilent Technologies). Sequencing was performed using the Illumina HiSeq 2500 platform (Illumina) at the IGA Technology Services (Udine).

Reads were processed using the miRPlant tool (An et al. 2014). At first, reads were trimmed for the adapter sequence, and the reads with a length lower than 18 and higher than 22 were discarded. The reads were aligned against the *A. thaliana* miRNAs in the miRBase database (http://mirbase.org/; Kozomara et al. 2019), with the Java‐coded bowtie algorithm implemented in miRPlant software not allowing any mismatch. For miRNA quantification, only reads completely covering a mature miRNA were considered. The differential miRNA expression analysis between the samples was performed using the edgeR package (Robinson et al. 2010). Read counts were normalized using the TMM normalization method implemented in the EDASeq package. Differentially expressed miRNAs were identified based on a False Discovery Rate‐corrected *P* value <0.05.

RNA‐Seq heatmap was generated using the R package heatmap.2. miRNA reads counts were z‐score transformed and clustered according to the Pearson correlation and “average linkage” method.

miRNA targets were identified on the DPMIND database (https://cbi.njau.edu.cn/DPMIND/; Fei et al. 2018), considering an expectation threshold of 3. GO term enrichment analysis of miRNA targets was performed using the Functional Annotation tool in DAVID (https://david.ncifcrf.gov/; Huang et al. 2009), applying a medium classification stringency.

### 2.4 Northern blot analysis

Small RNAs were purified from the previously collected samples using the mirPremier microRNA Isolation Kit (Sigma‐Aldrich). About 1.5 μg small RNAs was separated on a denaturing 15% polyacrylamide gel containing 7 M urea and then transferred on a Hybond‐N+ nylon membrane (GE Healthcare) in a semi‐dry electroblotter. DNA oligo probes were labeled with [γ‐^32^P]ATP using the mirVana Probe & Marker Kit (Thermo Fisher Scientific); probe sequences are reported in Table S1. Blots were pre‐hybridized for 30 min in the ULTRAHyb‐Oligo Hybridization Buffer (Thermo Fisher Scientific). Hybridization was performed at 38°C overnight, according to the manufacturer's instructions. Signals were detected on an autoradiography film. Blots were then stripped and rehybridized with a probe complementary to U6 (Table S1) as a loading control to overcome unintended differences in RNA loading. Band intensity was measured using the Image Lab software (Bio‐Rad). Accumulation of mature miRNAs was evaluated as the ratio between miRNA probe intensity and U6 probe intensity.

### 2.5 Real‐time RT‐PCR

Total RNAs were purified from the previously collected samples using the TRIzol reagent (Thermo Fisher Scientific), according to the manufacturer's instructions. After DNase treatment, first‐strand cDNA was synthesized from 2 μg of total RNA using the Superscript III Reverse Transcriptase Kit (Thermo Fisher Scientific). Real‐time RT‐PCR was performed using the Platinum SYBR Green qPCR SuperMix‐UDG kit (Thermo Fisher Scientific) and a StepOnePlus Real‐Time PCR System (Applied Biosystems). Each reaction (40 amplification cycles) was carried out in triplicate; melting curve analysis was applied to confirm amplification specificity. Primers for miRNAs and miRNA targets are listed in Table S2. Endogenous reference genes for data normalization were *β‐ACTIN* (At5g09810) and *UBIQUITIN 10* (At4g05320). Relative expression was evaluated using the 2^−ΔΔCT^ method (Livak & Schmittgen 2001).

### 2.6 Statistical analysis

Data in histograms are represented as mean ± *SD*. miRNA‐Seq data were compared by the edgeR package (Robinson et al. 2010) based on a False Discovery Rate‐corrected *P* value <0.05. Statistical significance of all other experimental data was evaluated using GraphPad Prism 7 (GraphPad Software); results were analyzed by one‐way ANOVA followed by a post hoc Tukey's test. Statistically significant variations at *P* < 0.05 are marked with letters, the same letter corresponding to nonstatistically significant differences.

## 3 RESULTS

### 3.1 *A. thaliana* and *A. halleri* do not show stress symptoms under the growth conditions applied

**FIGURE 1:**
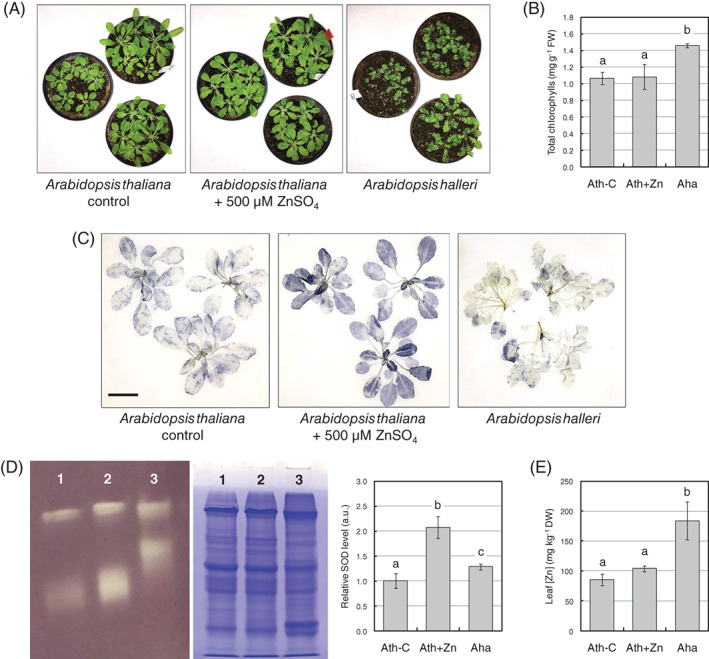
Physiological analysis of untreated (Ath‐C) and Zn‐treated *Arabidopsis thaliana* (Ath + Zn) and untreated *Arabidopsis halleri* (Aha) plants. (A) Phenotype of plants considered, at the end of the experiment. (B) Total chlorophyll content. (C) In situ O_2_^−^ visualization by NBT staining of whole rosettes. Bar corresponds to 3 cm. (D) Quantification of SOD activity, on 30 μg of total proteins: Left, in‐gel analysis of SOD activity; middle, Coomassie staining of the replica gel in SDS‐PAGE; right, relative quantification by densitometric analysis of SOD bands, normalized on total loading of SDS‐PAGE. Lanes: 1, untreated *A. thaliana*; 2, Zn‐treated *A. thaliana*; 3, *A. halleri*. (E) Zn concentration in leaves, as quantified by ICP‐AES. For histograms in B, D and E, statistically significant variations (*P* < 0.05) were evaluated by one‐way ANOVA followed by a post hoc Tukey's test and are marked with letters, the same letter corresponding to nonstatistically significant differences

*A. thaliana* plants were grown in soil upon control conditions (untreated soil) and moderate Zn enrichment (soil watered with 500 μM ZnSO_4_), whereas *A. halleri* was grown in control conditions to identify constitutively present strategies for Zn tolerance. To determine whether the growth conditions had an impact on plant fitness, the Zn accumulation and stress parameters were evaluated (Figure 1). All plants did not show visible stress symptoms and had a normal development (Figure 1A). Chlorophyll content was equal in untreated and Zn‐treated *A. thaliana* and about 40% higher in untreated *A. halleri* (Figure 1B); chlorophyll *a*/*b* ratio was similar in all plants considered (ca. 3.2, data not shown). NBT staining of whole rosettes revealed higher O_2_^−^ accumulation in Zn‐treated *A. thaliana* plants when compared with untreated *A. thaliana* and *A. halleri* ones (Figure 1C); this is associated with higher global SOD activity, as highlighted by in‐gel SOD analysis (Figure 1D). Zn accumulation was moderately, although not significantly, higher in Zn‐treated *A. thaliana* plants compared to untreated ones; Zn concentration in *A. halleri* leaves was double than in *A. thaliana* (Figure 1E).

### 3.2 Several miRNAs are differentially expressed under Zn treatment and between *A. thaliana and A. halleri*

**FIGURE 2:**
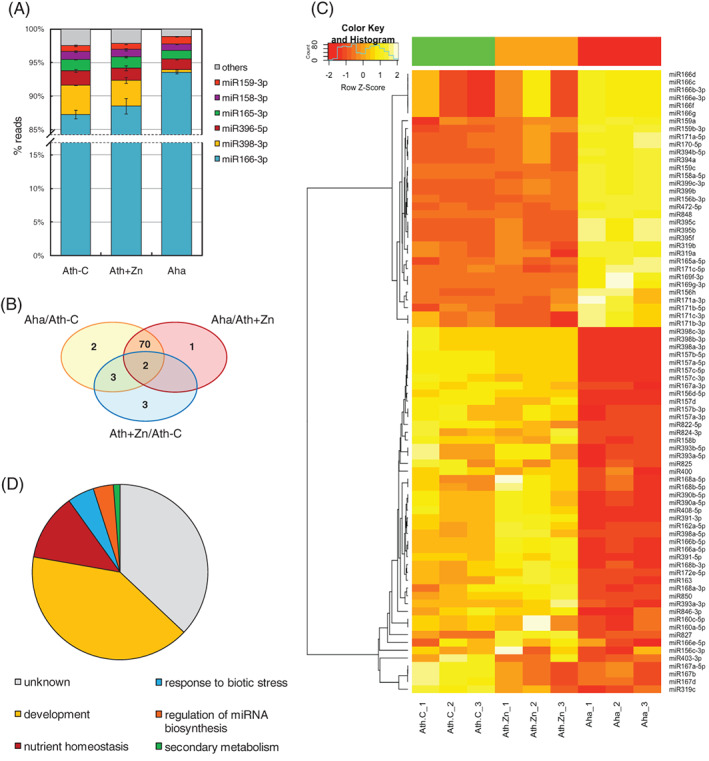
Identification and analysis of differentially expressed miRNAs by RNA‐Seq analysis, comparing untreated (Ath‐C) and Zn‐treated *A. thaliana* (Ath + Zn) and untreated *A. halleri* (Aha). (A) Most expressed miRNAs in the three sample groups; expression levels are represented as percentage of miRNA reads on total reads. (B) Venn diagram showing the number of differentially modulated miRNAs in the Aha/Ath‐C, Aha/Ath + Zn and Ath + Zn/Ath‐C comparisons. (C) Heatmap of all differentially modulated miRNAs; miRNA reads counts were z‐score transformed and clustered according to Pearson correlation and “average linkage” method. (D) Functional classification of differentially expressed miRNAs

The miRNA‐Seq analysis, considering untreated and Zn‐treated *A. thaliana* and untreated *A. halleri*, identified 129 expressed miRNAs for a total number of reads ranging between about 61 and 81 million/sample. The most represented family was miR166, which was also the most expressed in all genotypes and conditions (for 3p strands, 94% of all reads in *A. halleri*, about 88% in *A. thaliana*); also abundant were miR398, miR396, and miR165 isoforms (Figure 2A, Data S2). On the contrary, miR169 3p isoforms and miR395 were not expressed in *A. thaliana*. Moreover, no reads were detected for several miRNAs in *A. halleri*; of them, miR172e‐5p, miR391‐3p, and ‐5p showed complete sequence identity between *A. thaliana* and *A. halleri*, verified by sequence alignment with the *A. halleri* spp. *gemmifera* genome (Briskine et al. 2017), and therefore confirmed as not expressed. As for the other miRNAs not identified in *A. halleri*, they were either absent in the genome sequence or not conserved regarding sequence identity and were therefore not considered for further analysis.

Given these premises, the comparative analysis revealed 81 differentially expressed miRNAs belonging to 33 already described families (Table 1, Data S2). Major variations were found between *A. thaliana* and *A. halleri* (Figure 2B,C): 77 and 73 miRNAs were differentially expressed when comparing *A. halleri* with control and Zn‐treated *A. thaliana*, respectively. On the other hand, only eight miRNAs were significantly up‐ (miR163, miR398a‐5p, miR827, and miR850) or down‐regulated (miR167a‐5p, miR167b, miR167d, and miR822‐5p) upon Zn treatment in *A. thaliana*; miR163, miR167a‐5p, miR167b, miR167d, and miR827 were also down‐regulated in *A. halleri* compared to *A. thaliana*.

**TABLE 1:** List of differentially expressed miRNAs, as resulting from the comparative RNA‐Seq analysis between untreated (Ath‐C) and Zn‐treated *A. thaliana* (Ath + Zn) and untreated *A. halleri* (Aha)

Family	miRNA	FC [log_2_(Aha/Ath‐C)]	*P* value (Aha/Ath‐C)	FC [log_2_(Aha/Ath + Zn)]	*P* value (Aha/Ath + Zn)	FC [log_2_(Ath + Zn/Ath‐C)]	*P* value (Ath + Zn/Ath‐C)
miR156	ath‐miR156b‐3p	2.32	7.00E−27	2.12	9.68E−23		
	ath‐miR156c‐3p	−0.80	3.47E−02				
	ath‐miR156d‐5p	−1.23	2.29E−18	−1.10	3.71E−42		
	ath‐miR156h	2.09	2.82E−08	1.47	1.10E−05		
miR157	ath‐miR157a‐3p	−1.60	3.32E−12	−1.48	1.99E−08		
	ath‐miR157a‐5p	−2.69	3.85E−65	−2.54	2.49E−170		
	ath‐miR157b‐3p	−1.60	3.55E−12	−1.48	1.90E−08		
	ath‐miR157b‐5p	−2.69	1.95E−65	−2.54	1.07E−171		
	ath‐miR157c‐3p	−3.48	3.67E−92	−3.25	6.28E−143		
	ath‐miR157c‐5p	−2.69	1.79E−64	−2.54	1.45E−165		
	ath‐miR157d	−2.10	5.44E−38	−1.78	2.83E−42		
miR158	ath‐miR158a‐5p			3.38	4.30E−60		
	ath‐miR158b	−0.57	8.95E−04	−0.52	1.71E−03		
miR159	ath‐miR159a			0.30	9.58E−05		
	ath‐miR159b‐3p	0.45	2.58E−03	0.30	2.90E−04		
	ath‐miR159c	3.94	1.39E−140	3.93	2.04E−199		
miR160	ath‐miR160a‐5p	−0.64	1.28E–04	−0.73	2.04E−03		
	ath‐miR160c‐5p	−0.64	1.46E−04	−0.73	1.87E−03		
miR162	ath‐miR162a‐5p	−1.38	6.42E−12	−1.72	3.37E−16		
miR163	ath‐miR163	−1.53	2.77E−10	−2.19	4.94E−30	0.66	1.90E−03
miR165	ath‐miR165a‐5p	1.64	3.61E−10	1.58	2.69E−10		
miR166	ath‐miR166a‐5p	−1.45	2.27E−13	−1.76	1.86E−31		
	ath‐miR166b‐3p	0.35	3.47E−02	0.22	3.31E−02		
	ath‐miR166b‐5p	−1.45	1.71E−13	−1.76	1.18E−32		
	ath‐miR166c	0.35	3.47E−02	0.22	3.39E−02		
	ath‐miR166d	0.35	3.47E−02	0.22	3.58E−02		
	ath‐miR166e‐3p	0.35	3.61E−02	0.22	3.39E−02		
	ath‐miR166e‐5p			−0.99	9.64E−03		
	ath‐miR166f	0.35	3.49E−02	0.22	3.59E−02		
	ath‐miR166g	0.35	3.49E−02	0.22	3.31E−02		
miR167	ath‐miR167a‐3p	−1.48	1.00E−10	−1.38	8.08E−13		
	ath‐miR167a‐5p	−0.57	2.59E−03			−0.51	6.81E−05
	ath‐miR167b	−0.57	2.59E−03			−0.51	6.81E−05
	ath‐miR167d	−0.62	8.98E−04			−0.55	6.81E−05
miR168	ath‐miR168a‐3p	−0.54	1.74E−03	−0.77	2.34E−14		
	ath‐miR168a‐5p	−0.33	4.21E−02	−0.58	8.40E−08		
	ath‐miR168b‐3p	−0.79	1.02E−04	−1.07	8.24E−13		
	ath‐miR168b‐5p	−0.34	4.10E−02	−0.58	8.20E−08		
miR169	ath‐miR169f‐3p	OFF in Ath‐C	5.70E−46	OFF in Ath + Zn	4.79E−44		
	ath‐miR169g‐3p	OFF in Ath‐C	6.61E−22	OFF in Ath + Zn	1.24E−21		
miR170	ath‐miR170‐5p	1.38	5.51E−09	1.17	1.95E−06		
miR171	ath‐miR171a‐3p	2.62	7.16E−09	2.75	3.58E−08		
	ath‐miR171a‐5p	1.38	8.20E−09	1.17	2.48E−06		
	ath‐miR171b‐3p	0.56	1.90E−02	1.04	1.39E−05		
	ath‐miR171b‐5p	1.27	1.38E−03	1.20	2.09E−03		
	ath‐miR171c‐3p	0.56	1.71E−02	1.04	2.02E−05		
	ath‐miR171c‐5p	1.71	4.11E−06	2.67	1.12E−07		
miR172	ath‐miR172e‐5p	OFF in Aha	1.01E−25	OFF in Aha	5.96E−34		
miR319	ath‐miR319a	0.78	8.49E−06	0.85	1.42E−08		
	ath‐miR319b	0.97	8.40E−08	1.04	2.54E−11		
	ath‐miR319c	−0.70	5.34E−05				
miR390	ath‐miR390a‐5p	−1.39	7.16E−12	−1.55	1.20E−25		
	ath‐miR390b‐5p	−1.39	6.89E−12	−1.55	4.43E−26		
miR391	ath‐miR391‐3p	OFF in Aha	1.53E−77	OFF in Aha	1.14E−97		
	ath‐miR391‐5p	OFF in Aha	1.48E−116	OFF in Aha	9.78E−140		
miR393	ath‐miR393a‐3p	−2.66	6.20E−08	−3.03	3.20E−10		
	ath‐miR393a‐5p	−0.79	3.43E−05	−0.80	1.15E−06		
	ath‐miR393b‐5p	−0.79	4.42E−05	−0.80	1.69E−06		
miR394	ath‐miR394a	2.10	1.23E−15	1.55	1.68E−08		
	ath‐miR394b‐5p	2.12	5.47E−16	1.57	7.71E−09		
miR395	ath‐miR395b	OFF in Ath‐C	9.46E−22	4.18	1.34E−10		
	ath‐miR395c	OFF in Ath‐C	7.83E−22	4.18	2.94E−10		
	ath‐miR395f	OFF in Ath‐C	6.62E−22	4.18	1.45E−10		
miR398	ath‐miR398a‐3p	−3.30	2.07E−103	−3.21	0.00E+00		
	ath‐miR398a‐5p					0.62	4.17E−02
	ath‐miR398b‐3p	−3.31	2.63E−104	−3.21	0.00E+00		
	ath‐miR398c‐3p	−3.31	3.77E−103	−3.21	0.00E+00		
miR399	ath‐miR399b	2.71	3.64E−39	2.05	9.14E−32		
	ath‐miR399c‐3p	2.66	3.07E−38	2.06	1.86E−31		
miR400	ath‐miR400	−1.83	1.87E−03	−1.64	1.21E−02		
miR403	ath‐miR403‐3p	−0.60	1.58E−04	−0.31	1.89E−02		
miR408	ath‐miR408‐5p	−1.44	1.21E−17	−1.56	9.69E−63		
	ath‐miR408‐5p	−0.43	4.83E−02	−0.57	3.99E−02		
miR472	ath‐miR472‐5p	3.15	1.31E−47	2.89	1.14E−33		
miR822	ath‐miR822‐5p					−0.66	4.03E−02
miR824	ath‐miR824‐3p	−0.34	3.74E−02	−0.35	3.10E−03		
miR825	ath‐miR825	−2.31	1.48E−05	−2.05	4.22E−04		
miR827	ath‐miR827	−0.45	2.85E−02	−1.39	1.62E−13	0.94	5.08E−05
miR846	ath‐miR846‐3p	−0.98	4.55E−07	−1.01	3.72E−06		
miR848	ath‐miR848	3.48	1.30E−27	4.96	2.47E−37		
miR850	ath‐miR850					0.59	4.03E−02

Abbreviation: FC, fold‐change.

The function of a wide proportion (37%) of the modulated miRNAs is unknown: of these, the majority consists of the complementary sequences referenced as “passenger strands” and still poorly characterized. Of the remaining, the most represented functional class (41%) is associated with plant development; several miRNAs are also involved in nutrient homeostasis and response to biotic stresses (12% and 5%, respectively, Figure 2D).

**FIGURE 3:**
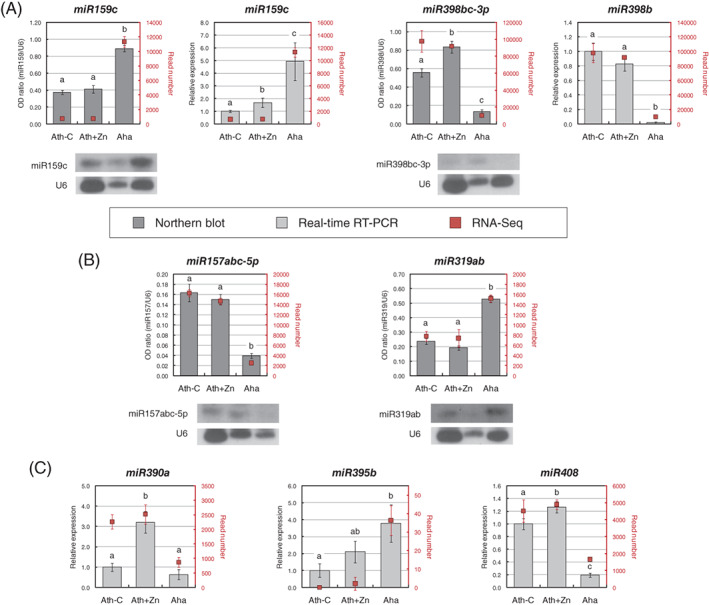
Validation of differentially modulated miRNAs. (A) Comparison of northern blot and real‐time RT‐PCR methods on miR159 and miR398. (B) Validation of miR157 and miR319 expression by northern blot. (C) Validation of miR390, miR395 and miR408 expression by real‐time RT‐PCR. Statistically significant variations (*P* < 0.05) in northern blot and real‐time RT‐PCR analyses, evaluated by one‐way ANOVA followed by a post hoc Tukey's test, are marked with letters, the same letter corresponding to nonstatistically significant differences

Validation of RNA‐Seq results was performed on a set of remarkable miRNAs involved either in development (miR157, miR159, miR319, and miR390) or nutrient homeostasis (miR395, miR398, and miR408). Northern blot on the mature miRNA or real‐time RT‐PCR on its precursor was applied according to their expression levels, estimated by their read counts in each sample in RNA‐Seq analysis. When low numbers of reads were detected, expression analysis by real‐time RT‐PCR on precursors was used. miR159 and miR398, having significantly high read counts, were tested by both methods to confirm the consistency of the analysis. The results confirmed the modulation of the selected miRNAs in untreated and Zn‐treated *A. thaliana*, and in untreated *A. halleri* (Figure 3).

### 3.3 Plant development and stress response are differentially controlled in *A. halleri* and upon Zn treatment in *A. thaliana*

miRNA targets were predicted by comparison with the DPMIND database (Fei et al. 2018); lists were manually integrated with data from the literature for those experimentally confirmed targets that were excluded by the bioinformatic prediction (Data S3). Functional annotation and enrichment analysis by the DAVID software produced several enriched functional clusters responsible for a variety of different molecular processes and biological functions (Data S4). When considering the targets of miRNAs modulated in *A. thaliana* upon Zn treatment, the functional analysis revealed the enrichment of S‐adenosylmethionine‐dependent methyltransferases (cluster 1, enrichment score [ES] = 10.05), involved in stress response and targeted by miR163. The other statistically significant enriched cluster under Zn treatment includes Zn finger and Cys/His‐rich proteins with unknown biological function (cluster 2, ES = 4.56), targeted by miR822. Regarding the comparison between *A. thaliana* and *A. halleri*, a wider set of functions was identified in miRNA targets. Stress response is enriched also in this target subset: in particular lectins (cluster 1, ES = 18.78) and tetra−/pentatricopeptide repeat proteins (cluster 3, ES = 7.20) are involved in defense against biotic stresses, whereas S‐adenosylmethionine‐dependent methyltransferases (cluster 4, ES = 5.72) and superoxide dismutases (cluster 7, ES = 2.99) are associated with more general stress responses. Moreover, functional enrichment revealed several clusters of transcription factors involved in various developmental processes (clusters 2, 6, 8, 9, 14, and 18, Data S4); of these, the most noticeable is associated with the auxin signaling pathway (cluster 8, ES = 2.25). Some enriched functions are also linked with mineral homeostasis, in particular Cu (clusters 11 and 16).

**FIGURE 4:**
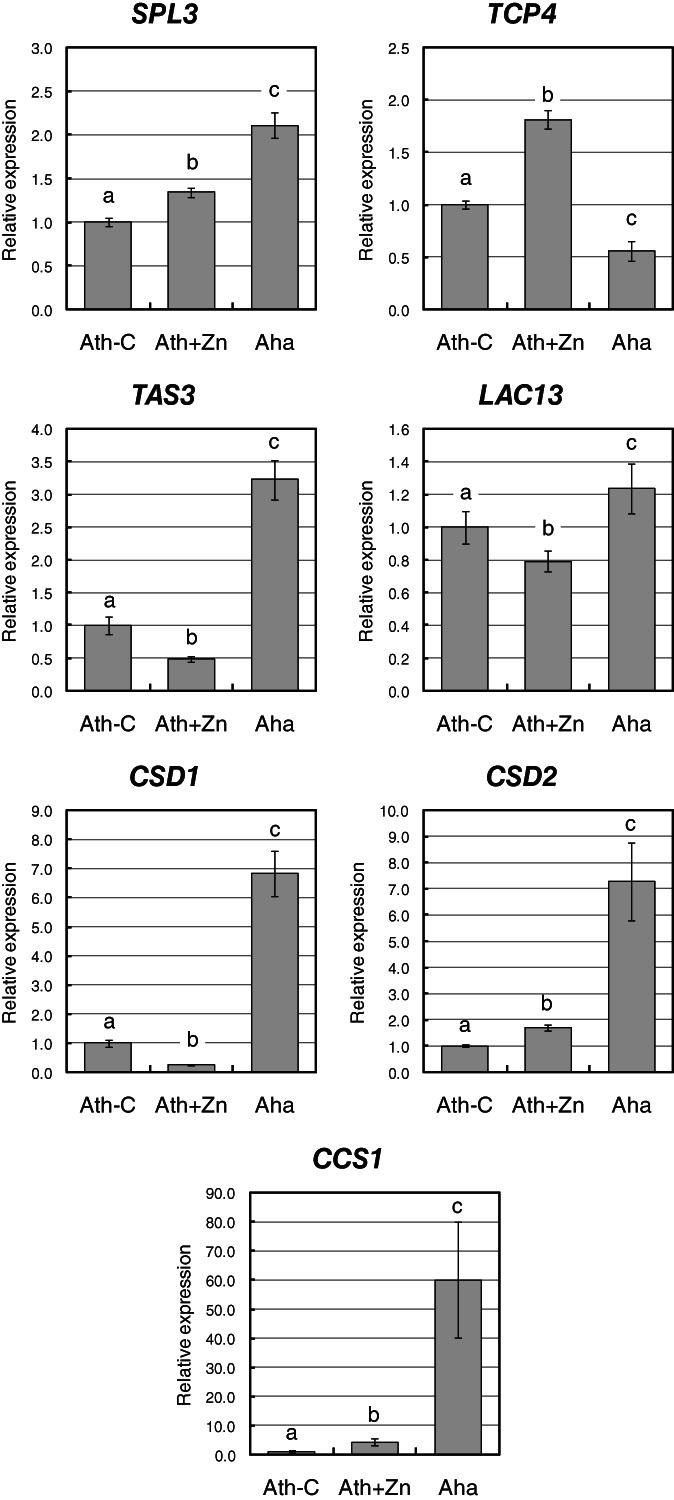
Expression analysis of miRNA targets, evaluated by real‐time RT‐PCR. Statistically significant variations (*P* < 0.05), evaluated by one‐way ANOVA followed by a post hoc Tukey's test, are marked with letters, the same letter corresponding to nonstatistically significant differences

To confirm the modulation of target genes, a real‐time RT‐PCR analysis was performed on untreated and Zn‐treated *A. thaliana* and on untreated *A. halleri*. The following targets were chosen due to their involvement in either development or stress response and to their regulation by cleavage: *SQUAMOSA PROMOTER BINDING PROTEIN‐LIKE 3* (*SPL3, AT2G33810*) for miR156 and miR157; *TCP FAMILY TRANSCRIPTION FACTOR 4* (*TCP4*, *AT3G15030*) for miR319; *TRANS‐ACTING SIRNA3* (*TAS3*, *AT3G17185*) for miR390; *COPPER/ZINC SUPEROXIDE DISMUTASE 1* (*CSD1*, *AT1G08830*), *COPPER/ZINC SUPEROXIDE DISMUTASE 2* (*CSD2*, *AT2G28190*), and *COPPER CHAPERONE FOR SOD1* (*CCS1*, *AT1G12520*) for miR398; and *LACCASE13* (*LAC13*, *AT5G07130*) for miR408 (Figure 4). Transcription factor *SPL3* was expressed at higher levels in *A. halleri*, in line with the overall lower levels of its regulators miR156‐5p and miR157‐5p. Analogously, the precursor of trans‐activated siRNA3, *TAS3*, the transcription factor *TCP4* has an expression profile that is consistent with its regulator. On the other hand, the transcript for laccase *LAC13* is only moderately up‐regulated in *A. halleri* in comparison to *A. thaliana*, as against a significantly low expression of miR408. Finally, the superoxide dismutases *CSD1* and *CSD2*, as well as *CCS1*, are significantly up‐regulated in *A. halleri* than in *A. thaliana*, in accordance to the expression levels of miR398; in response to Zn in *A. thaliana*, targets are either moderately up‐ (*CSD2* and *CCS1*) or down‐regulated (*CSD2*) in view of no significant modulation of miR398 (Figure 4).

## 4 DISCUSSION

miRNA involvement in the metal homeostasis of plants has been extensively documented (Ding et al. 2020; Noman & Aqeel 2017). However, scarce notice has been given to miRNAs in Zn homeostasis, except for some works considering Zn deficiency (Li et al. 2013; Shi et al. 2013). Due to its dual condition as an essential micronutrient and a toxic trace element when in excess (Andreini et al. 2009; DalCorso 2012), Zn uptake and distribution must be kept under strict control. This makes the analysis of plant response to Zn very interesting, although challenging: indeed, plant behavior toward this metal is extremely variable and associated with the adaptation to a wide range of different edaphic conditions. In particular, hyperaccumulator species show hypertolerance as well as tightly regulated uptake, translocation and compartmentalization of metals. Yet, despite the precise control of ionic balances displayed, no data are available on miRNAs in hyperaccumulators. In light of the evidence, this study has focused on miRNA involvement in Zn homeostasis by considering the effect of Zn excess on the nontolerant and nonaccumulator species *A. thaliana*, as well as strategies that are constitutively activated or repressed in the hyperaccumulator species *A. halleri*. Indeed, *A. halleri* has been reported to undergo constitutive Zn deficiency responses even under Zn‐sufficient conditions due to the extremely efficient metal translocation and compartmentalization (Hanikenne et al. 2008).

Under the tested conditions that were chosen in order not to induce excessive stress in nonaccumulator *A. thaliana*, Zn‐treated plants did not show apparent toxicity symptoms, apart from an increase in O_2_^−^ accumulation and in global SOD activity. Zn accumulation in leaves was slightly, but not significantly, higher, consistently with the characterization of *A. thaliana* as an excluder species (Arrivault et al. 2006). On the other hand, Zn accumulation in untreated *A. halleri* was significantly higher than in *A. thaliana*, although lower than what was observed in previous studies in native metallicolous soil and upon hydroponic conditions (Corso et al. 2021; Schvartzman et al. 2018). However, in this study, plants were grown in unpolluted soil, having low Zn content and bioavailability. This considered, it is possible that *A. halleri* was under moderate Zn deficiency, but the condition was not so substantial as to produce an appreciable phenotype of Zn deprivation, as evidenced by the physiological characterization.

The most striking result emerging from miRNA‐Seq analysis is that a consistent number of miRNAs is differentially expressed when comparing the two species considered, whereas a significantly smaller subset is modulated upon Zn treatment in *A. thaliana*. Analogously, Zn deficiency in *S. bicolor* produced only a small set of modulated miRNAs in leaves, despite a significant reduction in plant growth (Li et al. 2013). Moreover, it must be remembered that the Zn treatment applied in this study produced a condition of moderately Zn‐rich soil in order to not induce excessive stress in *A. thaliana*. Interestingly, miRNAs that are modulated by Zn treatment in *A. thaliana* are also differentially expressed when comparing *A. thaliana* with hyperaccumulator *A. halleri*, supporting the hypothesis of a constitutive activation/repression of specific processes in the latter. With this in mind, the stress response is significantly enriched in the targets of Zn‐regulated miRNAs. For example, miR163 and miR827, both up‐regulated under Zn treatment in *A. thaliana* and underexpressed in *A. halleri*, have been reported as involved in macronutrient imbalance (Kant et al. 2011; Lundmark et al. 2010) and biotic stress (Chow & Ng 2017; Yaeno & Iba 2008). Very low levels of miR163 expression had been previously found also in *Arabidopsis arenosa* (Ng et al. 2011), a pseudo‐metallophyte evolved independently from *A. halleri*, coherently with the hypothesis of partially convergent adaptive processes in the behavior toward metals (Preite et al. 2019). Interestingly, some other miRNAs involved in defense against biotic stresses were found as constitutively expressed at different levels in *A. halleri* in comparison to *A. thaliana* (e.g., miR400, miR472, miR825, and miR846). This evidence is coherent with the evolution of different defense mechanisms in *A. halleri* by changes in copy number and expression levels of biotic stress‐related genes (Becher et al. 2004; Suryawanshi et al. 2016). Indeed, this adaptive strategy is the result of both convergence between response strategies against biotic/abiotic stresses and metal hyperaccumulation, providing some form of elemental defense against pathogens (Shahzad et al. 2013; Stolpe et al. 2017).

In addition to the stress‐related miRNAs reported above, a substantially large group of differentially expressed miRNAs emerge from the comparison between *A. thaliana* and *A. halleri*, likely reflecting the differences in the developmental plan and nutritional strategies between the two species. Indeed, among the predicted targets of differentially expressed miRNAs, those associated with plant growth are significantly enriched; among them, genes responsible for the development of roots, leaves and floral organs are represented. To explain this, it must be considered that the two species, despite being phylogenetically close, vary in terms of life history (*A. thaliana* is annual, whereas *A. halleri* is perennial and stoloniferous) and therefore have different allocation of vegetative versus reproductive growth (Clauss & Koch 2006; Honjo & Kudoh 2019). However, plant habitus is likely not the only factor contributing to miRNA differential expression. In fact, growth and development are strongly associated with specific strategies for nutrient uptake and distribution and respond to elemental imbalances in the soil (Bonser & Aarssen 2003; Bonser et al. 2010); moreover, miRNAs regulating developmental genes constitute a sizeable subset of those modulated under nutrient deficiencies or metal excess (Chien et al. 2017; Noman & Aqeel 2017). In particular, root growth and architecture are highly responsive to element availability in the soil (Forde & Lorenzo 2001); although the analyses conducted in this study focus on miRNA regulation in leaves, there is ample proof for the control of root development and functioning through mobile signals produced by the shoot, such as phytohormones and small RNAs (Chuck & O'Connor 2010; Puig et al. 2012). Given all the evidence reported above, the variability in miRNA levels between *A. thaliana* and *A. halleri* mirrors their inherent differences both in developmental plans and in nutrient requirements and strategies for their acquisition. In particular, miRNAs directly involved in auxin signaling, such as miR160, miR167, and miR393 (Mallory et al. 2005; Si‐Ammour et al. 2011; Wu et al. 2006), constitute a notable and consistent subgroup of those differentially expressed between the two species. Moreover, also miR319 and miR390 participate in auxin regulation, respectively, by targeting *TCP* transcription factors, among which *TCP4* that controls *YUCCA5* flavin monooxygenase involved in auxin biosynthesis (Challa et al. 2016), and by inducing the maturation of *TAS3* tasiRNA, a *trans*‐acting small regulatory RNA repressing the expression of *ARF3* (Fahlgren et al. 2006; Montgomery et al. 2008). It should be noticed that these miRNAs have been implicated in phenotypic plasticity in response to nutrient deficiency (Liang et al. 2012; Vidal et al. 2010), stress (Iglesias et al. 2014; He et al. 2018), and other environmental cues. Interestingly, miR167‐5p isoforms, expressed at higher levels in *A. thaliana* grown in control conditions in comparison with both *A. thaliana* upon Zn treatment and *A. halleri*, were proposed to be down‐regulated by Cd in *Brassica napus* and to target the metal transporter *BnNRAMP1b*, located in the plasma membrane and able to transport Zn, Cd, and Mn (Meng et al. 2017). Although *AtNRAMP1* was not identified by bioinformatic analysis among miR167 targets in *A. thaliana* in this study, possible involvement of this regulatory RNA in the control of metal transport cannot be excluded.

Besides miRNAs associated with plant development, the comparison between *A. thaliana* and *A. halleri* highlighted the modulation of a notable range of miRNAs involved in nutrition. Among them, some P‐related miRNAs are differentially expressed in *A. halleri*, including the above‐cited miR163 and miR827 as well as miR399, a key regulator of P homeostasis (Pant et al. 2008). Indeed, the association between Zn and P nutrition has been extensively reported (reviewed in Bouain et al. 2014). Furthermore, miR395 isoforms, involved in the regulation of S homeostasis, have been detected only in the pseudo‐metallophyte species, although transcript levels are low. Under S starvation, miR395 targets two ATP sulfurylases, *ATPS1* and *ATPS4*, as well as *SULTR2;1*, a low‐affinity sulfate transporter, allowing a more efficient redistribution of S to the shoot; in conditions of S sufficiency, miR395 is not expressed, thus explaining the absence of the transcript in *A. thaliana* and the low read numbers in *A. halleri* (Kawashima et al. 2011; Liang et al. 2010). On the other hand, miR395 differential expression in *A. halleri* may be linked with different nutritional needs of the pseudo‐metallophyte; indeed, sulfur metabolism is integrated in the complex network controlling the homeostasis of both macronutrients and trace elements (Briat et al. 2015; Na & Salt 2011).

Finally, in line with the different profiles of mineral nutrition that distinguish the two species under analysis, Cu‐responsive miR398 and miR408 are significantly down‐regulated in *A. halleri* when compared with *A. thaliana*. These two miRNAs are involved in the redistribution of Cu resources and the maintaining of Cu homeostasis under Cu deficiency by targeting nonessential Cu‐binding proteins (reviewed by Pilon 2017). In particular, miR398‐3p targets the Cu/Zn superoxide dismutases *CSD1* and *CSD2*, with cytoplasmic and plastidial localization, respectively (Sunkar et al. 2006), as well as the associated Cu chaperone *CCS1* (Beauclair et al. 2010). In this view, miR398 links mineral homeostasis with the control of oxidative stress. Indeed, the lower expression of miR398‐3p in *A. halleri* is consistent with the constitutively higher expression of the targets *CSD1*, *CSD2* and *CCS1* observed in this study and the lower accumulation of reactive oxygen species already described in the pseudo‐metallophyte (Baliardini et al. 2015; Chiang et al. 2006). On the other hand, miR398‐3p isoforms were not modulated upon Zn treatment in *A. thaliana*. This is apparently in contrasts with the higher O_2_^−^ accumulation and SOD levels observed in Zn‐treated *A. thaliana* in this study and with miR398 down‐regulation upon oxidative stress and excess of redox‐active metals (Sunkar et al. 2006). However, Zn is not a directly redox‐active metal (Cuypers et al. 1999), and it has been proposed to alter redox homeostasis indirectly, with no significant effect on expression levels of miR398b and c (Remans et al. 2012). As for their targets, *CSD1* expression was down‐regulated in the same conditions, whereas *CSD2* and *CCS1* were moderately but significantly induced, and global SOD levels are almost double in Zn‐treated plants than in control conditions. Cu/Zn SOD modulation was contrary to what was observed by Remans et al. (2012); however, it should be considered that the treatment imposed in this study is different in both the growth substrate and the duration, thus resulting in a milder stress. Interestingly, by the miRNA‐Seq analysis, only miR398a‐5p was up‐regulated upon Zn treatment in *A. thaliana*. This isoform belongs to the poorly characterized passenger strands; the targets predicted in this study do not allow a clear definition of its possible role in the plant, although the drought‐inducible transcription factor *ERF053* has been proposed as a putative target (Zhu et al. 2020). Overall, Zn treatment upon sub‐toxic conditions determines a moderate alteration of redox status in *A. thaliana*, that correlates with the modulation of SOD genes but not with that of miR398. On the other hand, the expression of the whole regulatory hub associated with miR398 is markedly different in *A. halleri*, leading to constitutively activated strategies for defense against oxidative stress as a part of the adaptive background of metal hypertolerance.

In conclusion, in *A. thaliana*, high Zn in soil induces the modulation of a small set of miRNAs involved in stress response, nutrition and plant development that are constitutively down‐regulated in the facultative metallophyte *A. halleri*. In addition to these, several other miRNAs have substantially different transcript levels in *A. halleri* than *A. thaliana*, coherently with native differences in development and nutrient homeostasis, as well as with constitutively activated strategies for stress response, in particular for oxidative stress. Overall, these results support the hypothesis that adaptation to metalliferous soils implicates the reorganization of plant growth, allocation of resources and global mineral nutrition.

## AUTHOR CONTRIBUTIONS

Antonella Furini, Giovanni DalCorso, and Elisa Fasani conceived the project; Antonella Furini and Giovanni DalCorso supervised the study. Elisa Fasani, Giovanni DalCorso, and Gianluca Zorzi performed the experiments, whereas Nicola Vitulo attended to the curation of RNA‐Seq data; Elisa Fasani, Giovanni DalCorso, and Nicola Vitulo contributed to the interpretation of data. Elisa Fasani wrote the draft of the manuscript; Giovanni DalCorso, Antonella Furini, and Nicola Vitulo provided revisions and editing.

## Supporting information


**DATA S1** List of probes for Northern blot analysis of mature miRNAs (Table S1) and of primers for real‐time RT‐PCR analysis of pre‐miRNAs and targets (Table S2).

**DATA S2** Results of the RNA‐Seq analysis on miRNAs in *A. thaliana* under control conditions (Ath‐C) and upon Zn treatment (Ath + Zn) and in *A. halleri* under control conditions (Aha). Statistically significant differences are in bold.

**DATA S3** List of miRNA targets as predicted by comparison with the DPMIND database and by literature.

**DATA S4** List of clusters from the functional enrichment analysis.

## Data Availability

All data supporting the findings of this study are available within the paper and within its supplementary materials published online.
