# Supplementary material for: Comparative analysis identifies micro‐RNA associated with nutrient homeostasis, development and stress response in Arabidopsis thaliana upon high Zn and metal hyperaccumulator Arabidopsis halleri
Source: Physiol Plant. 2021 Jul 5;173(3):920–34. doi: 10.1111/ppl.13488 (PMC8597110; doi:10.1111/ppl.13488)
Supplement: Supplementary file 1 — DATA S1 List of probes for Northern blot analysis of mature miRNAs (Table S1) and of primers for real‐time RT‐PCR analysis of pre‐miRNAs and targets (Table S2). [file PPL-173-920-s003.docx]

**Table S1:** List of DNA oligo probes for Northern blot analysis of miRNA expression.

| **Target miRNAs** | **Probe sequence** |
| --- | --- |
| miR157a-5p, miR157b-5p, miR157c-5p | 5’-GTGCTCTCTATCTTCTGTCAA-3’ |
| miR159c | 5’-AGGAGCTCCCTTCAATCCAAA-3’ |
| miR319a, miR319b | 5’-AGGGAGCTCCCTTCAGTCCAA-3’ |
| miR398b-3p, miR398c-3p | 5’-TGTGTTCTCAGGTCACCCCTG-3’ |
| snRNA U6 | 5’-TCATCCTTGCGCAGGGGCCA-3’ |

**Table S2:** List of primers for real-time RT-PCR analysis.

| **Type of transcript** | **Transcript** | **Primer sequences** |
| --- | --- | --- |
| miRNA precursors | *MIR159c* | Fw: 5’-GAGGACAAGATTAGAGGAACT-3’  Rev: 5’-CAATCCAAACGAAGAGAAGAG-3’ |
|  | *MIR390a* | Fw: 5’-GTAGAGAAGAATCTGTAAAGCT-3’  Rev: 5’-AGTAAGAAGAGCCAATGAAACT-3’ |
|  | *MIR395b* | Fw: 5’-CTTTCAAATTCCCACATGTTCTT-3’  Rev: 5’-ACTTCAGTGTTATATACAAACCAA-3’ |
|  | *MIR398b* | Fw: 5’-TGAAGGTAGTGGATCTCGAC-3’  Rev: 5’- GGGTGACCTGAGAACACATG-3’ |
|  | *MIR408* | Fw: 5’-GGCCAATTTCAAAGGTTAGATT-3’  Rev: 5’-TTCAGCTCCACTTCATGAATG-3’ |
| miRNA targets | *SPL3* | Fw: 5’-CCAAACAGTACCACAAACGAC-3’  Rev: 5’-GCTTGGCTTCATCAAACTCAC-3’ |
|  | *TCP4* | Fw: 5’-GGTTTCAGGGCCAAGAAGAG-3’  Rev: 5’-AAGTCAAGCTTCAATGTGCATG-3’ |
|  | *TAS3* | Fw: 5’-CTTGTAAGGCCTTTTCTTGAC-3’  Rev: 5’-GGGATAGACAAGGTAGGAGA-3’ |
|  | *CSD1* | Fw: 5’-TCCATGCAGACCCTGATGA-3’  Rev: 5’-TTAGCCCTGGAGACCAATGA-3’ |
|  | *CSD2* | Fw: 5’-GAGCCTTTGTGGTTCACGAG-3’  Rev: 5’-CCAATCACACCACATGCCAA-3’ |
|  | *CCS1* | Fw: 5’-AACGAGTATGGAGATCTCACA-3’  Rev: 5’-TTGTCTGCCTCTAGTGTTCC-3’ |
|  | *LAC13* | Fw: 5’- AAGAAACACAATCGGAACACC-3’  Rev: 5’- TTGCTAAACCCCAGAATATATG-3’ |
